# Utility of Scanning Electron Microscopy Elemental Analysis Using the ‘NanoSuit’ Correlative Light and Electron Microscopy Method in the Diagnosis of Lanthanum Phosphate Deposition in the Esophagogastroduodenal Mucosa

**DOI:** 10.3390/diagnostics10010001

**Published:** 2019-12-18

**Authors:** Kazuya Shinmura, Hideya Kawasaki, Satoshi Baba, Isao Ohta, Hisami Kato, Hideo Yasuda, Satoshi Yamada, Kiyoshi Misawa, Ken Sugimoto, Satoshi Osawa, Masanori Sato, Takahiko Hariyama, Haruhiko Sugimura

**Affiliations:** 1 Department of Tumor Pathology, Hamamatsu University School of Medicine, Hamamatsu, Shizuoka 431-3192, Japan; hisami@hama-med.ac.jp (H.K.); hsugimur@hama-med.ac.jp (H.S.); 2 Institute for NanoSuit Research, Preeminent Medical Photonics Education & Research Center, Hamamatsu University School of Medicine, Hamamatsu, Shizuoka 431-3192, Japan; hariyama@hama-med.ac.jp; 3 Department of Diagnostic Pathology, Hamamatsu University School of Medicine, Hamamatsu, Shizuoka 431-3192, Japan; baba@hama-med.ac.jp; 4 Advanced Research Facilities and Services, Preeminent Medical Photonics Education & Research Center, Hamamatsu University School of Medicine, Hamamatsu, Shizuoka 431-3192, Japan; ohtaisao@hama-med.ac.jp; 5 First Department of Medicine, Hamamatsu University School of Medicine, Hamamatsu, Shizuoka 431-3192, Japan; ysdh@hama-med.ac.jp (H.Y.); sugimken@hama-med.ac.jp (K.S.); 6 Department of Otolaryngology/Head and Neck Surgery, Hamamatsu University School of Medicine, Hamamatsu, Shizuoka 431-3192, Japan; 41240280@hama-med.ac.jp (S.Y.); kiyoshim@hama-med.ac.jp (K.M.); 7 Department of Endoscopic and Photodynamic Medicine, Hamamatsu University School of Medicine, Hamamatsu, Shizuoka 431-3192, Japan; sososawa@hama-med.ac.jp; 8 First Department of Surgery, Hamamatsu University School of Medicine, Hamamatsu, Shizuoka 431-3192, Japan; msnrsato@hama-med.ac.jp

**Keywords:** lanthanum phosphate deposition, correlative light and electron microscopy (CLEM), NanoSuit-CLEM method, scanning electron microscopy (SEM)-energy dispersive X-ray spectroscopy (EDS), elemental analysis

## Abstract

Background: We have recently developed the correlative light and electron microscopy of hematoxylin and eosin (H&E)-stained glass slides using the ‘NanoSuit’ method. The aim of this study is to explore the utility of the new NanoSuit-correlative light and electron microscopy method combined with scanning electron microscopy-energy dispersive X-ray spectroscopy elemental analysis for the diagnosis of lanthanum phosphate deposition in the H&E-stained glass slides. Methods: Nine H&E-stained glass slides of the upper gastrointestinal tract mucosa containing the brown pigmented areas by light microscopic observation, which were suspected as lanthanum phosphate deposition, were observed and analyzed by scanning electron microscopy-energy dispersive X-ray spectroscopy using the NanoSuit-correlative light and electron microscopy method. Results: In all nine slides, the new NanoSuit-correlative light and electron microscopy method combined with scanning electron microscopy-energy dispersive X-ray spectroscopy revealed the accumulation of both lanthanum and phosphorus in the tissue area corresponding to the brown pigment deposition. In addition to the existence of lanthanum phosphate in the stomach and duodenum, known target organs, we observed deposition in the esophagus for the first time. Furthermore, we observed lanthanum phosphate deposition in the background mucosa of stomach containing primary adenocarcinoma. Conclusions: Scanning electron microscopy-energy dispersive X-ray spectroscopy analysis using the NanoSuit-correlative light and electron microscopy method is useful for the diagnosis of lanthanum phosphate deposition in the H&E-stained glass slides. Lanthanum phosphate deposition occurs not only in the stomach and duodenum but also in the esophagus.

## 1. Introduction

Lanthanum carbonate is one of the therapeutic phosphate binders used to keep optimal serum phosphate levels in dialysis patients with end-stage renal disease [1]. Lanthanum carbonate binds with ingested phosphate to form insoluble complexes in the digestive tract, which are excreted with the feces [2]. However, recent reports have reported that small amounts of lanthanum phosphate are deposited in the stomach and duodenum in patients treated with lanthanum carbonate [3,4,5,6,7,8,9,10,11,12,13,14,15]. The lanthanum phosphate deposition is frequently observed especially in the stomach [6,14], and is suggested to be associated with some pathological conditions such as intestinal metaplasia and foveolar hyperplasia [5,7,8,14]. Cases that show both primary adenocarcinoma and lanthanum phosphate deposition in the stomach have also been reported [3,5,7,11,14,15]. However, whether lanthanum phosphate deposition causes gastric adenocarcinoma has not been elucidated. In a rat model of oral administration of lanthanum carbonate, gastric mucosal alterations such as intestinal metaplasia, epithelial hyperplasia, and proliferation of mucosal neck cells were reported [14]. Regarding the *Helicobacter pylori* infection status in humans, lanthanum phosphate deposition occurs irrespective of the infection status [8,11]. As various pathological lesions, including malignant neoplasia, can occur in the stomach and duodenum, differential diagnosis of lanthanum phosphate deposition from these pathological lesions is important. In light microscopic observation with hematoxylin and eosin (H&E) staining, the deposits appear as a brown granular, needle-shaped, or amorphous substance within and outside the histiocytes [9,12]. Sometimes their colors are described as eosinophilic, grey, or purple [3,7,9,13]. It is important to diagnose definitively whether the deposits showing several different shapes or colors are lanthanum phosphate or not using element analysis such as an energy dispersive X-ray spectroscopy (EDS) combined with a scanning electron microscopy (SEM) [11,13]. However, preparation of samples for the traditional SEM-EDS requires several complicated procedures, using at least two different pathological slides and sputtering metal particles, such as osmium tetroxide, which is harmful to humans [16]. Therefore, element analysis is rarely performed and the deposits are diagnosed only based on the slight differences observed under a light microscope in routine pathological diagnosis.

We previously reported that polymerization of a natural extracellular substance on the outer surface of animals by electron beam or plasma irradiation resulted in the formation of a nano-scale layer, called the ‘NanoSuit’, which enables small animals to stay alive under SEM observation [17]. We have recently developed the new correlative light and electron microscopy (CLEM) method using NanoSuit (NanoSuit-CLEM method), which enables not only CLEM but also EDS analysis [18,19]. The sections of formalin-fixed paraffin-embedded (FFPE) tissue on glass slides are easily observed both by light and electron microscope showing less structural damage and weight loss than in the case of conventional methods for SEM observation [18,19]. This method requires only several minutes to observe the pathological slides after the cover slips are removed, and does not require additional treatment, such as osmium conductive metal coating.

Here, we explore the utility of the NanoSuit-CLEM method for the elemental analysis of the deposition, which is suspected as lanthanum phosphate, observed in the H&E-stained FFPE tissue section. Moreover, we tried to find lanthanum phosphate deposition in organs of the digestive tract, other than stomach and duodenum, which are already known as lanthanum phosphate-deposited organs.

## 2. Materials and Methods

### 2.1. Patient Collection

Nine H&E-stained FFPE slides of the upper gastrointestinal tract mucosa tissue containing the brown pigmented area by light microscopic observation, which were suspected as lanthanum phosphate deposition in routine pathological diagnosis, were used in this study. All nine slides were derived from six patients and were obtained at Hamamatsu University Hospital (HUH) in Japan (Table 1). All of them had a history of dialysis and lanthanum carbonate treatment (Table 1). This study was approved by the Institutional Review Board of Hamamatsu University School of Medicine (reference number: 18-074, approval date: 29 June 2018) and this study was carried out in accordance with the World Medical Association Declaration of Helsinki.

### 2.2. NanoSuit-CLEM Method Combined with SEM-EDS

**Figure 1:**
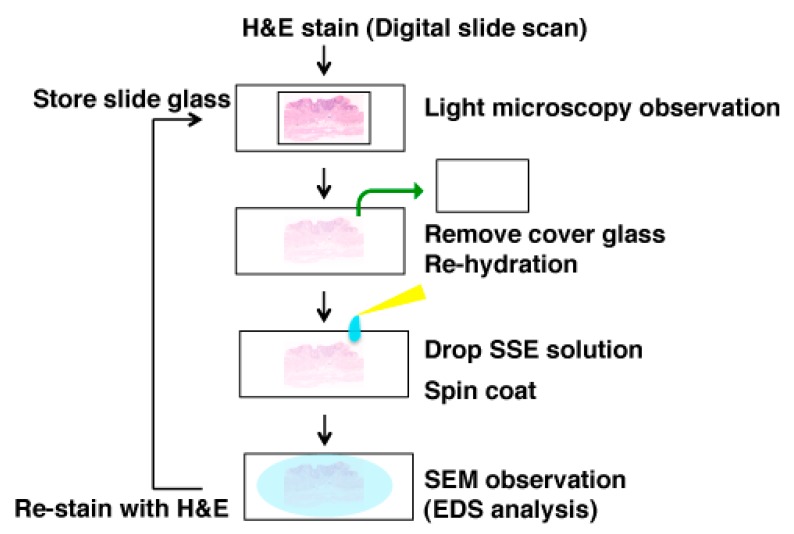
Scheme of the scanning electron microscopy (SEM) analysis using the NanoSuit-correlative light and electron microscopy (CLEM) method. If pathologists find any pathological substance, which should be analyzed at a higher resolution in an H&E slide, of which an image can be stored by the digital pathology system, during light microscopic observation, the tissue slide can be used for SEM observation. After removal of the cover glass and rehydration of the tissue, by coating surface shield enhancer (SSE) solution on the tissue, the slide is ready for SEM observation. SEM-energy dispersive X-ray spectroscopy (EDS) analysis is also available. After SEM observation, re-staining with H&E enables us to store the slide. If exact original H&E images are needed, use of the digital pathology system (also called whole slide imaging system) [20], before SEM-EDS analysis, would be helpful.

The brown pigments on the slides in the light microscopic examination were investigated by SEM observation using the NanoSuit-CLEM method as shown in Figure 1. First, the cover glass was removed with xylene and the section on the slide was re-hydrated using a drop of surface shield enhancer (SSE) solution. The stock solution of SSE consisted of sucrose, fructose, and sodium chloride dissolved in distilled water, to which citric acid and sodium glutamate (pH 7.4) were then added [18]. The resulting aqueous solution was mixed with glycerine at a ratio of 1:2. SSE was diluted 20-fold in ethanol. Sections were subsequently spin-coated by centrifugation at 2000 rpm for 15 sec to remove excess of the SSE solution [18]. The specimens were then directly introduced into the SEM to form the NanoSuit following irradiation of the electron beam. Elemental analysis, including lanthanum and phosphorus, was performed with SEM (TM4000Plus; HITACHI, Tokyo, Japan; accelerating voltage: 15 kV) equipped with an EDS (X-stream-2; Oxford instruments, Oxford, UK). AZtecOne software (Oxford instruments) was used for the EDS analysis. The weight concentrations of all detected elements were quantified and expressed as weight percentage (wt%), which shows the relative concentration of an element in the analyzed area. After ultrastructural observation, the specimens were re-stained with H&E and the cover glass was mounted with diaphane (Malinol, Muto Pure Chemicals, Tokyo, Japan) for storage.

### 2.3. Statistical Analysis

The wt% values were statistically analyzed using the Mann–Whitney *U* test (SAS Institute, Cary, NC, USA).

## 3. Results

### 3.1. NanoSuit-CLEM Method Combined with SEM-EDS Is Useful for the Diagnosis of Lanthanum Phosphate Deposition in the H&E-Stained Glass Slides

**Figure 2:**
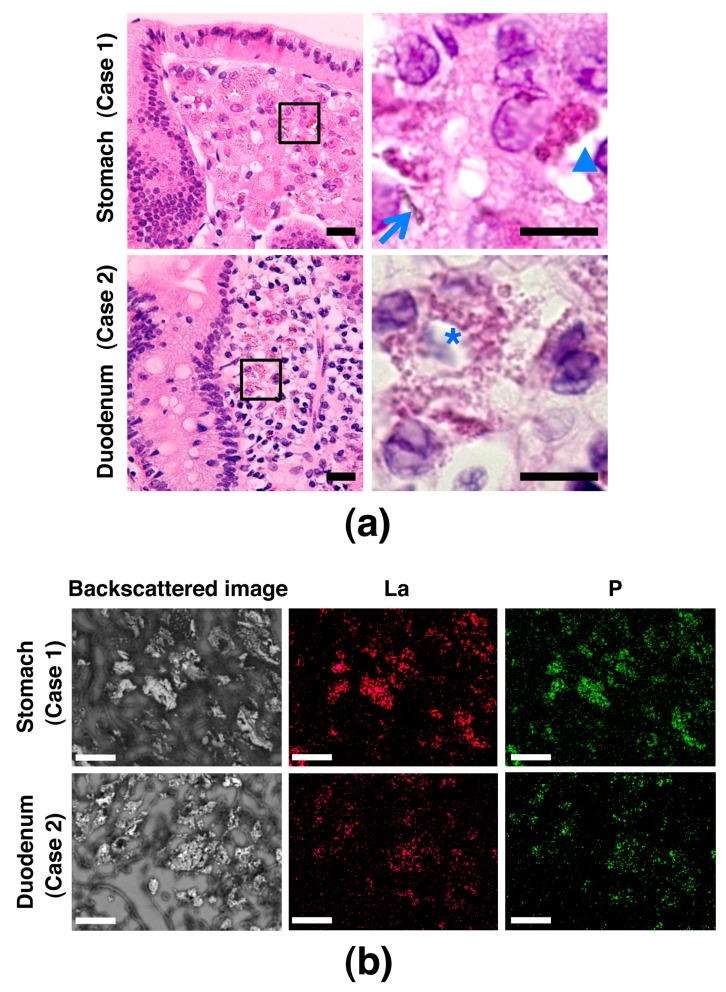
Lanthanum phosphate deposition in the stomach and duodenum. (**a**) Images by light microscopy of H&E-stained gastric and duodenal mucosa containing brown pigment deposition, which was suspected as lanthanum phosphate deposition. Images in the right column are a higher magnification of the boxed areas in the images in the left column. Depositions showing granular, needle-shaped, or amorphous structures are marked with an asterisk, arrow, or arrowhead, respectively. Scale bar = 20 μm (left); 10 μm (right). (**b**) Lanthanum phosphate deposition in the gastric and duodenal mucosa shown by SEM-EDS analysis using the NanoSuit-CLEM method. Images in the left column are backscattered SEM images showing a bright area in the mucosa. The middle- and right-column images are elemental mapping images using SEM-EDS analysis showing deposition of lanthanum (La) and phosphorus (P), respectively. Scale bar = 25 μm.

**Figure 3:**
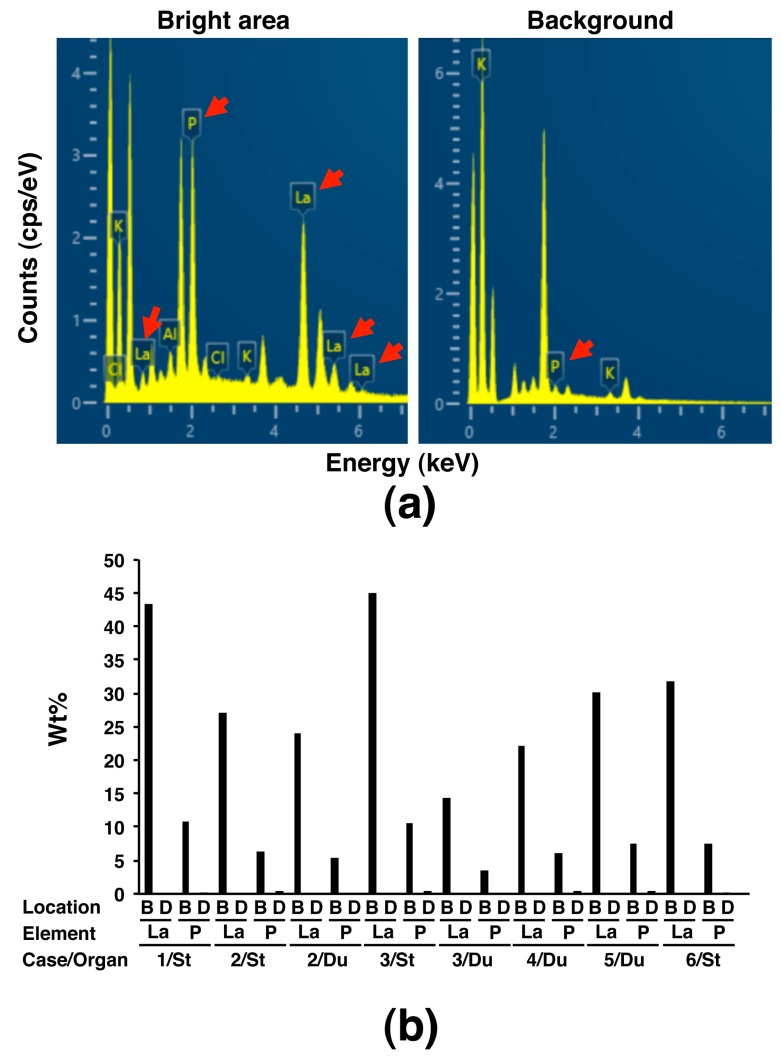
Spectrum analysis based on the SEM-EDS using the NanoSuit-CLEM method of substances suspected as lanthanum phosphate deposition in the gastric and duodenal mucosa. (**a**) Spectrum in the bright area (left) and background (right) by SEM (case 3/stomach). The arrow indicates peak of lanthanum (La) or phosphorus (P). (**b**) Comparison of weight percentage (wt%) of La or P obtained by spectrum analysis between the bright area (B) and background at other dimmer area (D) in eight slides. St, stomach; Du, duodenum.

To explore whether SEM observation using the NanoSuit-CLEM method is useful for the diagnosis of lanthanum phosphate deposition in the gastrointestinal tract, we first performed SEM observation in six cases (cases 1–6) having a pathological record of brown pigment deposition, which were suspected as lanthanum phosphate deposition, in the H&E slides, either or both of stomach and duodenum (Figure 2a; representative images showing deposition exhibiting granular, needle-shaped, or amorphous structure), known as target organs of lanthanum phosphate deposition [8,12]. The same area was observed with the aid of the NanoSuit-CLEM method. The area where brown pigment deposition consisting of granular, needle-shaped, and/or amorphous structures was observed under a light microscope showed bright contrast in backscattered images, which were the images of incident electrons reflected back from the specimens, using SEM in all the eight slides derived from six cases (Figure 2b, Supplementary Figure S1). Further elemental mapping by SEM-EDS revealed the localization of lanthanum (La) and phosphorus (P) in the bright area through the backscattered images (Figure 2b, Supplementary Figure S1). Moreover, comparative analysis of the EDS spectrum showed that the counts (cps/eV) both of La and P in the bright area were much higher than those in the background in other dimmer mucosal area (Figure 3a); the weight percentage (wt%) values were significantly higher in the bright area than in the background (*p* < 0.001 for both La and P; Figure 3b; Wt% values of elements other than La and P in the analysis were summarized in Supplementary Table S1). These results indicated that the brown pigment deposition under light microscope, corresponding to the bright area in an SEM, was identical to lanthanum phosphate deposition in this experiment. Since previous literature reported the existence of adenocarcinoma in the stomach where lanthanum phosphate deposition was observed [3,5,7,11,14,15], we also examined whether there is a history of gastric or duodenal adenocarcinoma in our cases. Then we found that one case (case 6) was affected by early-stage gastric adenocarcinoma (Supplementary Figure S2) and lanthanum phosphate was deposited in the stomach (Supplementary Figure S1), meaning it is an applicable case.

### 3.2. Detection of Lanthanum Phosphate Deposition in the Esophagus

**Figure 4:**
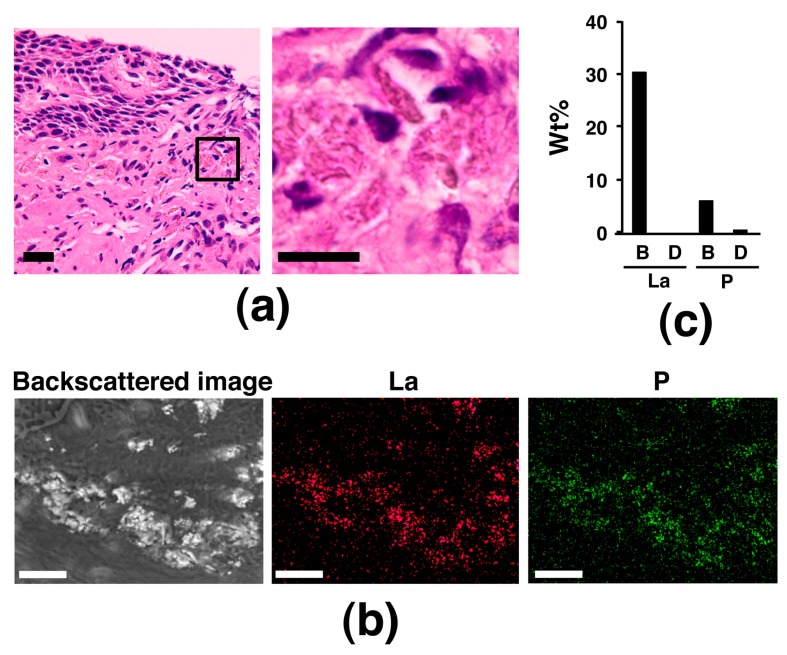
Lanthanum phosphate deposition in the esophageal mucosa. (**a**) Images by light microscopy of H&E-stained esophageal mucosa containing brown pigment deposition, which was suspected as lanthanum phosphate deposition. The image in the right panel is a higher magnification of the boxed area in the image in the left panel. Scale bar = 20 μm (left); 10 μm (right). (**b**) SEM-EDS analysis using the NanoSuit-CLEM method for brown pigment deposition in the esophageal mucosa. Results of elemental mapping for La and P are shown. Scale bar = 25 μm. (**c**) Spectrum analysis based on SEM-EDS using the NanoSuit-CLEM method. The wt% of La or P obtained by spectrum analysis was compared between the bright area (B) and the background at other dimmer area (D).

Next, we paid attention to the gastrointestinal tract organs, other than the stomach and duodenum, as a target of lanthanum phosphate deposition. Since four of the above six cases also underwent colon biopsy, we examined these biopsy specimens; however, no lanthanum phosphate deposition was observed. Next, since there was a record of a brown pigment deposition area in the biopsy specimen of the esophagus, which is believed not to be a target organ of lanthanum phosphate deposition, in case 6, the area was also examined with the NanoSuit-CLEM method combined with EDS. Surprisingly, the area where brown pigment deposition was observed under light microscope was shown bright in backscattered image in a SEM observation in case 6 and elemental mapping by SEM-EDS revealed that La and P in the bright area and EDS spectrum analysis showed high peaks of La and P in the bright area (Figure 4), indicating brown pigment deposition in the esophagus was identical to lanthanum phosphate deposition. When the clinical history of case 6 was more profoundly examined, two and a half years before the esophageal biopsy, the patient underwent proximal gastrectomy with esophagus-residual stomach anastomosis because of gastric adenocarcinoma, opening the possibility that the operation might be related with lanthanum phosphate deposition in the esophagus.

### 3.3. Restoring of the Slides Used for the NanoSuit-CLEM Method Combined with EDS

**Figure 5:**
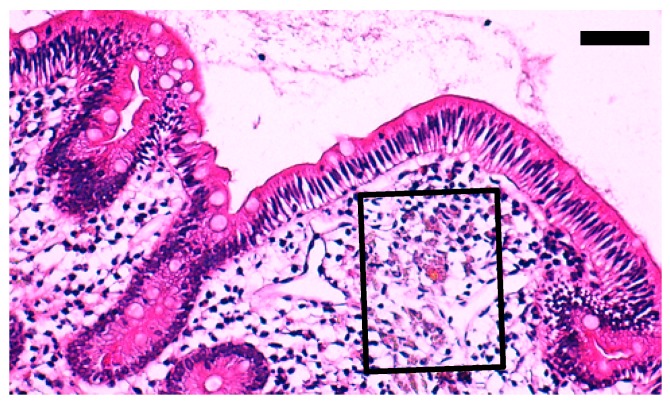
Representative result of re-staining with H&E after SEM observation (case 2, duodenum). Boxed area is the area used for elemental mapping analysis based on SEM-EDS using the NanoSuit-CLEM method. Scale bar = 50 μm. Another representative result is shown in Supplementary Figure S3.

After SEM observation using the NanoSuit-CLEM method combined with EDS, we examined the condition of the slides for restoring. Since slides became clearer after rehydration, we tried to re-stain them by H&E solution, and the staining condition of the areas used for element mapping was compared with the areas not used for it. The results showed that the condition was almost unchanged in the staining intensity between the two kinds of areas (Figure 5, Supplementary Figure S3), meaning that the NanoSuit-CLEM method combined with EDS seems not to damage the slides significantly, therefore, this method is suitable for the analysis by pathologists.

## 4. Discussion

In the present study, we showed that La and P deposition in the esophagogastroduodenal mucosa analyzed with our new NanoSuit-CLEM method combined with SEM-EDS, suggesting that the method is effective for the diagnosis of lanthanum phosphate deposition in the H&E-stained FFPE tissue sections. Therefore, we proposed performing SEM-EDS analysis using the NanoSuit-CLEM method when brown pigment deposition, which is suspected to be lanthanum phosphate deposition, is seen in H&E slides in routine pathological diagnoses. This would contribute to making a definite diagnosis. The following are considered to be the advantages of using the NanoSuit-CLEM method, combined with SEM-EDS, which we developed. (1) Although it is difficult to diagnose lanthanum phosphate depositions based only on light microscope observation because of their various shapes and colors, the NanoSuit-CLEM method combined with SEM-EDS enables us to diagnose it easily. (2) Not using osmium tetroxide, a hazardous chemical [16], which is an indispensable reagent in conventional SEM analysis. (3) Capability of using H&E slides, which are prepared for routine pathological diagnosis. This means that samples for SEM-EDS analysis can be prepared quickly and the possibility of loss of target substance due to the use of different slide sets, which are newly prepared after light microscopic examination, is avoided. (4) Imaging occurs in a wet state approximating natural conditions. Less structural damage has been proven using the NanoSuit-CLEM method compared to the conventional method for SEM observation [18,19].

The present study showed, for the first time, that lanthanum phosphate was deposited in the esophagus, apart from the stomach and duodenum, both of which are known target organs [8,12]. This result indicated that lanthanum phosphate penetrates through not only columnar epithelium, which is the surface epithelium of the stomach and duodenum, but also stratified squamous epithelium, which is the surface epithelium of the esophagus, providing us with the novel and interesting knowledge of the in vivo kinetics of lanthanum. However, caution may be required. Lanthanum phosphate deposition in the esophagus was detected only in case 6, and before the detection the case underwent proximal gastrectomy with esophagus-residual stomach anastomosis due to gastric adenocarcinoma in the gastric cardia. Therefore, it is speculated that such structural change in the upper gastrointestinal tract might result in the promotion of lanthanum phosphate deposition. Future accumulation of cases with esophageal lanthanum phosphate deposition would allow for clarification of its cause more solidly. In addition, investigating whether lanthanum phosphate is deposited in the gastrointestinal tract, other than the esophagus, stomach, or duodenum, would be important, since they all are exposed to orally administrated lanthanum carbonate. Judging from the result of our present study and the result of a previous study [11], it seems that the colon is not a main target organ of lanthanum phosphate deposition. Further studies will help our understanding of the organs in which lanthanum phosphate deposition occurs, and its relationship with disease. Interestingly, case 6 had gastric adenocarcinoma, as mentioned above. So far, several reports have documented cases affected with both primary adenocarcinoma and lanthanum phosphate deposition in the stomach [3,5,7,11,14,15]. Although the pathogenic effect of lanthanum phosphate deposition on gastric adenocarcinoma is unclear at present, future long-term observations of whether neoplastic change occurs in the stomach showing lanthanum phosphate deposition would elucidate the relationship between both factors.

The NanoSuit-CLEM method combined with SEM-EDS is available for the analysis of other depositions in human tissues. We have very recently evaluated patients with siderosis [19]. SEM-EDS analysis clearly revealed the expected ferrous deposition, and some of the iron deposits may comprise phosphorous iron oxide. Anthracosis of the lung was analyzed as well. The SEM-EDS analysis revealed the presence of multiple elements (Al, Si, Mg, O, and C) in lung tissue sections and that some heavy metals depositions were oxidized. Schimeca et al. [21] summarized that EDS microanalysis using SEM-EDS or transmission electron microscopy (TEM)-EDS could represent a good tool for (i) investigating the accumulation of heavy metals, (ii) characterizing a different isotype of calcification, (iii) studying the toxic effect and potential drug delivery of nanoparticles, and (iv) identifying a specific asbestos isotype in asbestos-related disease. According to the above-mentioned lists (i–iv), EDS analysis using the NanoSuit-CLEM method could become a powerful tool for pathological diagnostic applications.

Recent advances in the technologies for electron microscopy have led to the production of the desktop/tabletop electron microscope [22,23]. The NanoSuit-CLEM method combined with SEM-EDS can be used in several types of SEM, including field emission (FE)-SEM, and the convenient and affordable equipment might be suitable for medical practice. The combination of desktop/tabletop electron microscope and the NanoSuit-CLEM method, together with SEM-EDS, we developed for H&E-stained slides, would extend the possibility of using electron microscopes in clinical practice for pathological diagnosis.

## Figures and Tables

**Table 1:** Case characteristics.

Case No.	Sex	Age (Years) ^1^	Deposited Organ ^2^	Dialysis Vintage (Years)	Length of Lanthanum Carbonate Treatment (Years, Months)	Other Pathological Findings
1	Female	68	St	20	5 y, 7 m	-
2	Male	60	St, Du	32	3 y, 4 m	Gastric intestinal metaplasia
3	Male	68	St, Du	4	2 y, 6 m	-
4	Male	77	Du	7	2 y, 9 m	-
5	Male	51	Du	7	1 y, 1 m	-
6	Male	76	St, Es	21	5 y, 1 m	Gastric adenocarcinoma

^1^ Age at which brown pigment deposition, which was suspected as lanthanum phosphate deposition, was first found in the esophagogastroduodenal mucosa. ^2^ St, stomach; Du, duodenum; Es, esophagus.

## Supplementary Materials

The following are available online at https://www.mdpi.com/2075-4418/10/1/1/s1, Table S1: List of weight percentage (wt%) of elements detected by SEM-EDS analysis using the NanoSuit-CLEM method, Figure S1: The other results detecting lanthanum phosphate deposition in the gastroduodenal mucosa by SEM-EDS analysis using the NanoSuit-CLEM method (cases 2–6), Figure S2: Images by light microscopy of H&E-stained primary adenocarcinoma in the stomach containing lanthanum phosphate deposition (case 6), Figure S3: Another representative result of re-staining with H&E after SEM observation (case 2, stomach).
