# Supplementary material for: Utility of Scanning Electron Microscopy Elemental Analysis Using the ‘NanoSuit’ Correlative Light and Electron Microscopy Method in the Diagnosis of Lanthanum Phosphate Deposition in the Esophagogastroduodenal Mucosa
Source: Diagnostics (Basel). 2019 Dec 18;10(1):1. doi: 10.3390/diagnostics10010001 (PMC7167806; doi:10.3390/diagnostics10010001)
Supplement: Supplementary file 1 [file diagnostics-10-00001-s001.pdf]

Supplementary Material

Supplementary Table S1. List of weight percentage (wt%) of elements detected by SEM-EDS analysis using the NanoSuit-CLEM method.

Case	Organ [1]	Location [2]	Element [3]									
			La	P	C	O	Si	Ca	Na	Br	S	Al
No.1	St	B	43.3	10.8	<0.1	29.6	7.6	2.3	2.3	1.5	0.6	<0.1
		D	<0.1	0.2	46.9	20.5	21.7	4.3	3.5	<0.1	0.2	0.7
No.2	St	B	27.0	6.4	24.5	30.0	7.6	1.9	1.7	<0.1	<0.1	0.5
		D	<0.1	0.4	62.1	22.0	9.7	1.9	1.7	0.9	0.3	<0.1
No.2	Du	B	24.0	5.4	32.0	29.4	5.5	1.5	1.4	<0.1	0.2	0.4
		D	<0.1	<0.1	67.2	14.9	12.3	2.5	1.4	0.7	<0.1	<0.1
No.3	St	B	45.1	10.5	<0.1	26.5	9.2	2.8	2.5	<0.1	1.1	1.2
		D	<0.1	0.3	65.3	22.4	7.5	1.3	1.3	0.8	0.4	<0.1
No.3	Du	B	14.4	3.5	40.7	23.3	10.7	2.2	2.2	<0.1	0.4	0.7
		D	<0.1	0.1	57.6	19.7	13.9	2.6	2.7	<0.1	0.3	0.6
No.4	Du	B	22.0	6.0	32.3	27.6	6.9	2.1	1.5	1.3	<0.1	<0.1
		D	<0.1	0.3	59.9	16.3	15.7	3.5	1.8	<0.1	0.5	0.6
No.5	Du	B	30.2	7.6	21.5	27.3	7.3	2.4	1.9	1.3	0.3	<0.1
		D	<0.1	0.4	65.6	19.9	8.7	1.6	1.7	<0.1	0.6	0.5
No.6	St	B	30.4	6.1	36.1	19.7	4.0	1.5	1.0	<0.1	0.5	0.5
		D	<0.1	0.3	57.5	28.2	8.4	1.4	2.2	0.7	0.4	<0.1
No.6	Es	B	31.9	7.6	24.0	25.5	5.8	1.9	1.5	1.3	0.2	<0.1
		D	<0.1	0.2	60.7	14.8	15.7	3.5	1.8	<0.1	0.3	0.6

[1] St, stomach; Du, duodenum; Es, esophagus. [2] B, bright area; D, background at other dimmer area. [3] If wt% ≥1.0% was detected in at least one measurement, the element is listed in this table.

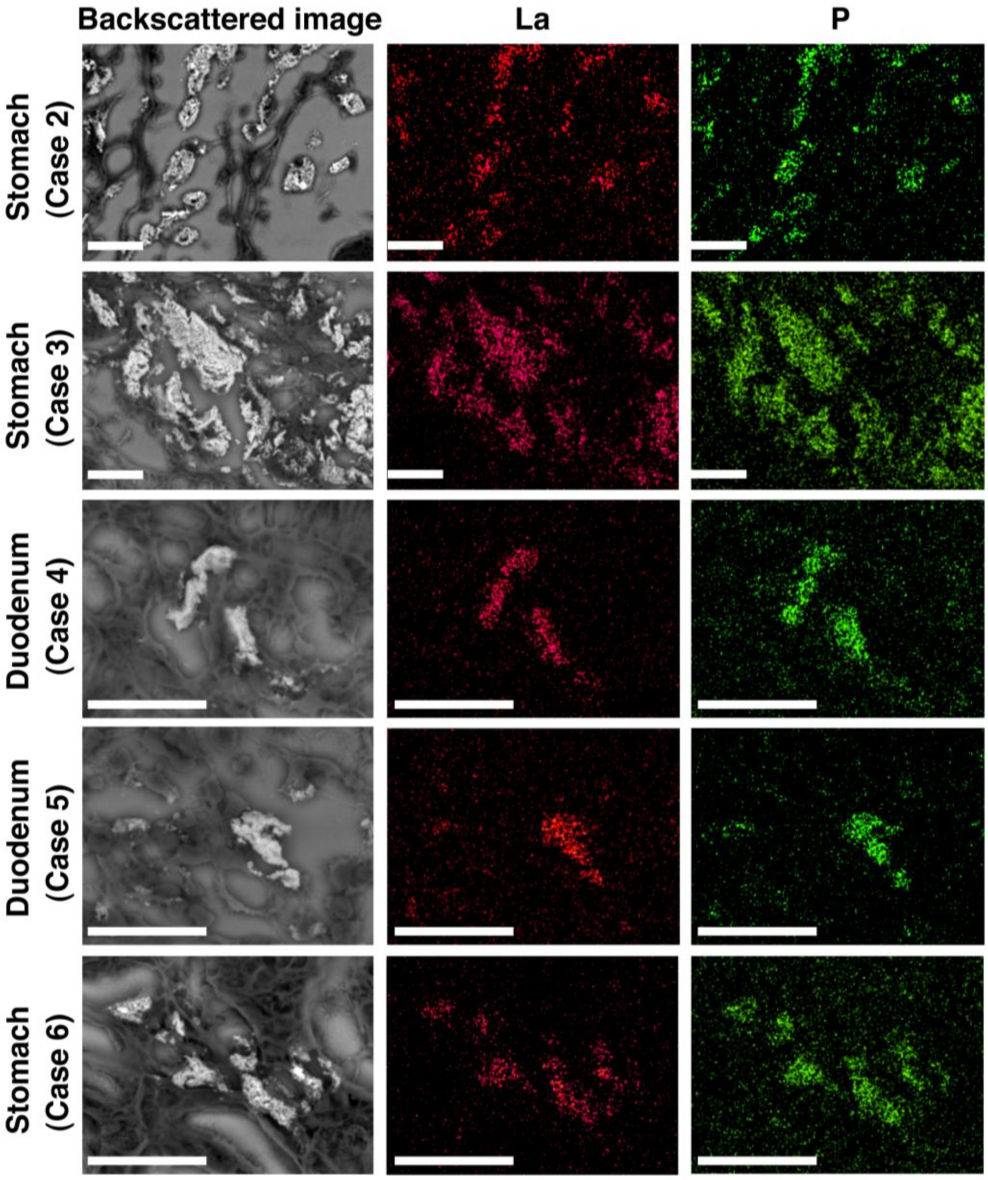

Supplementary Figure S1. The other results detecting lanthanum phosphate deposition in the gastroduodenal mucosa by SEM-EDS analysis using the NanoSuit-CLEM method (cases 2–6). The results other than the results shown in Figure 2 are shown in this figure. Images in the left column are backscattered SEM images showing a bright area in the mucosa. The middle- and right-column images are elemental mapping images using SEM-EDS analysis showing deposition of lanthanum (La) and phosphorus (P), respectively. Scale bar = 25 m.

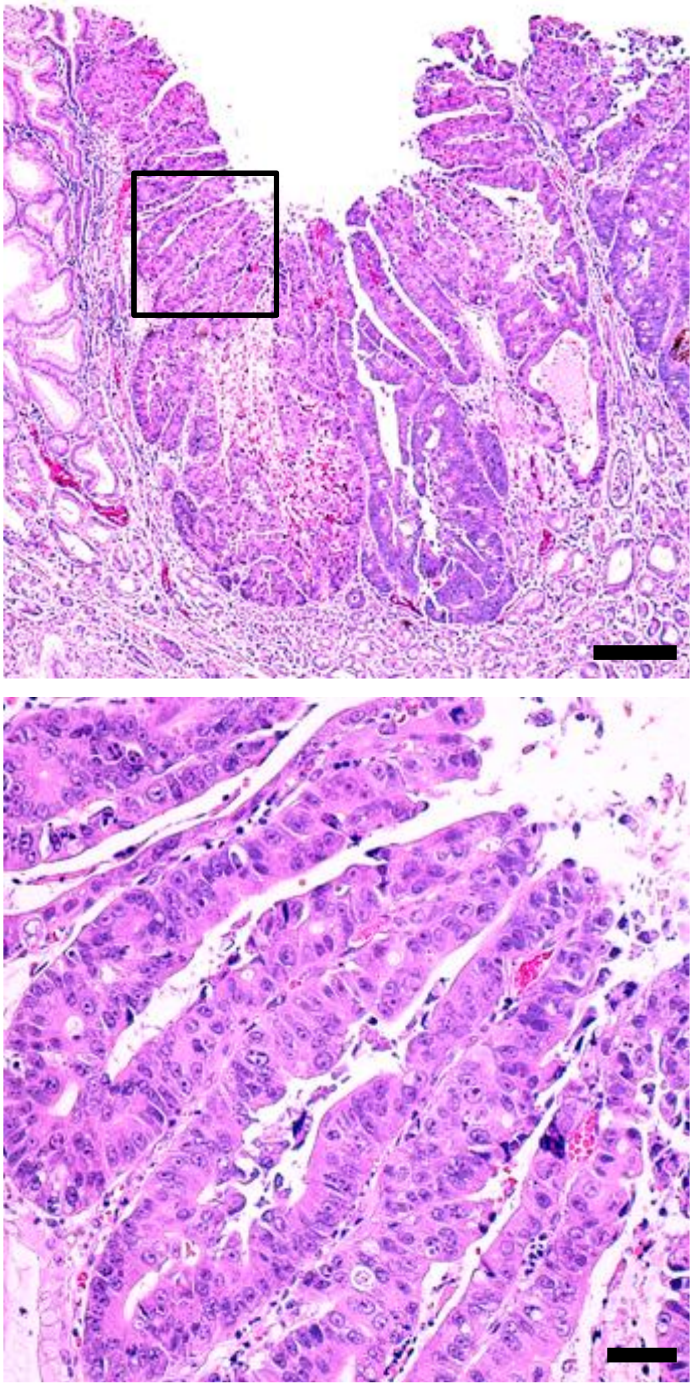

Supplementary Figure S2. Images by light microscopy of H&E-stained primary adenocarcinoma in the stomach containing lanthanum phosphate deposition (case 6). Image in the lower panel is a higher magnification of the boxed area in the image in the upper panel. Scale bar = 200 m (upper); 40 m (lower)..

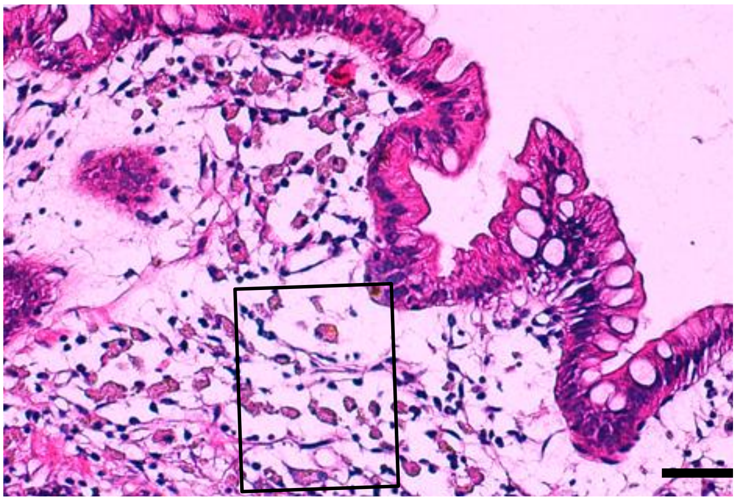

Supplementary Figure S3. Another representative result of re-staining with H&E after SEM observation (case 2, stomach). Boxed area is the area used for elemental mapping analysis based on SEM-EDS using the NanoSuit-CLEM method. Scale bar = 50 m.
